# Antenatal Depression and Associated Factors among Pregnant Women Attending Antenatal Care Service in Kochi Health Center, Jimma Town, Ethiopia

**DOI:** 10.1155/2021/5047432

**Published:** 2021-02-08

**Authors:** Yonas Tesfaye, Liyew Agenagnew

**Affiliations:** Department of Psychiatry, Faculty of Medical Sciences, Institute of Health, Jimma University, Jimma, Ethiopia

## Abstract

**Background:**

Antenatal depression has immense public health importance, as it can adversely affect both the mother and child health. The problem contributes to the disease burden in both developed and developing countries. Despite this, it is less investigated and not getting the necessary attention in the study setting.

**Objective:**

The aim of the study was to assess the prevalence of antenatal depression and associated factors among women attending antenatal care (ANC) service in Kochi Health Center, Jimma town, southwest Ethiopia, 2019.

**Method:**

Institutional based cross-sectional survey was conducted on 314 pregnant women attending Kochi Health Center from February 15 to April 15, 2019. A systematic random sampling technique was used to include the study participants. Antenatal depression was assessed using the Patient Health Questionnaire (PHQ-9) tool. Data was collected through face-to-face interviews using a pretested and structured questionnaire. Descriptive statistics was done to summarize the dependent and independent variables. Moreover, the chi-square test analysis was done to determine the association between the outcome and explanatory variables.

**Results:**

A total of 314 pregnant women participated in the study, making a response rate of 96.7%. The study has revealed a total of 52 (16.6%) of the respondent had antenatal depression. A chi-square test of independence analysis showed a significant association between antenatal depression and marital status, family history of depression, pregnancy planning, history of abortion, social support, and intimate partner violence (*P* < 0.00001).

**Conclusion:**

The study has shown that the prevalence of antenatal depression was high and associated with multiple psychosocial, clinical, and obstetric factors. Therefore, screening pregnant women for depression and the provision of necessary mental health services is recommended to mitigate the adverse health outcome of the problem.

## 1. Introduction

Globally, more than 350 million people of all ages suffer from depression, and more women are affected by depression than men [1, 2]. According to the World Health Organization (WHO), depression is projected to become the second leading contributor to the global burden of disease by 2020 [3].

Common mental disorders such as depression are one of the public health problems of the developing world. Studies have shown that depression and substance abuse are rampant, overwhelmingly disabling, follows chronic courses when untreated, and can lead to increased morbidity and mortality [4, 5].

Depressed women are more likely to have unhealthy practices during pregnancy, poor nutrition due to a lack of appetite, poor weight gain, and risk for intrauterine growth. Depressed women are less compliant with prenatal care and feel less invested in the care toward their pregnancy [6–8].

Antenatal depression is a condition that describes a range of physical and emotional changes that many women can have during pregnancy. Some of these symptoms include fatigue, crying, hopelessness, anxiety, problems concentrating, changes in appetite, sleeping difficulty, lack of interest in daily activities, and suicide. It is estimated that 25-35% of pregnant women have depressive symptoms, and 20% of them may meet the diagnostic criteria for major depression at some time during pregnancy [9, 10].

It is a multifaceted illness that has varying consequences for a woman's mental health, physical health, the family's functioning, and her children's [11–13]. Antenatal depressive symptoms have the potential to impact negatively upon health service utilization and thereby contribute to increased perinatal complications, adverse pregnancy outcomes, and maternal mortality [14, 15].

Untreated depression during pregnancy has been also associated with poor pregnancy and birth outcomes such as maternal preeclampsia, low birth weight, smaller head circumference, increased risk of premature delivery, increased surgical delivery interventions, and lower Appearance, Pulse, Grimace, Activity, and Respiration(APGAR) scores and is considered to be the strongest risk factor for postpartum depression [16–18].

In the developing world, antenatal depression is a critical public health problem because of its intergenerational impact on the mother, infants, and children [19]. One in three to one in five women in developing countries has a significant mental health problem during pregnancy and after childbirth [20, 21]. In terms of regional distributions, high rates of mental health problems in pregnant women and mothers have been reported from Africa [22].

Many studies which have done in the topic area in Ethiopia were from the different regions of the country, having the knowledge of socio-cultural-economic difference among the regions in the country; this study will give an insight towards antenatal depression and associated factor among pregnant women attending ANC service in Jimma town, southwest Ethiopia. Despite national and international organizations focus on maternal mental health, antenatal depression remains a neglected issue in healthcare systems and given low priority in the study setting. Moreover, there is a paucity of evidence on antenatal depression. Indeed, better evidence is needed to support greater priority to mental health care and to design appropriate interventions. Additionally, the findings of this study will serve as a baseline for further research in the area.

## 2. Materials and Methods

### 2.1. Study Area and Period

Kochi Health Center is located in Jimma town, which is found in Oromia regional state, southwest of Ethiopia, 352 km far away from the capital city, Addis Ababa. The health center is owned by the government and serving almost ten kebeles (lowest administrative unit) in the town. The health center has started health service in December 2000 and provides services such as outpatient treatment, ANC service, maternal and child health service, delivery service, antiretroviral therapy service, HIV/AIDS prevention from mother to child service, and public health services with an outreach program for a total of 11503 population in the catchment area. During the study, there were 1287 pregnant women who had ANC follow-up in the health center. The study was conducted from February 15 to April 15, 2019.

### 2.2. Study Design and Population

The institution-based cross-sectional study design was conducted on randomly sampled pregnant women attending ANC services during the study period.

### 2.3. Sample Size Determination

The sample size was computed through a single population proportion formula, *n* = [(*Z* *α*/2)^2^ *p* (1 − *p*)]/*d*^2^, by assuming the prevalence of antenatal depression among pregnant woman rate 50% to get the maximum sample size, 5% margin of error, and 95% confidence level of certainty (alpha = 0.05). Accordingly, *n* = (1.96)^2^ × 0.5(1 − 0.5)/(0.05)^2^ = 384. Since the total population is less than ten thousand, finite population correction formula was used to get the desired sample size. Hence, nf = *n*/(1 + *n*/*N*); hence, nf = 384/(1 + 384/1287) = 295. Finally, with the addition of a 10% nonresponse rate, the total sample size was 325.

### 2.4. Sampling Technique and Procedure

A systematic random sampling technique was used to include pregnant women attending the Kochi Health Center ANC clinic during the data collection period. The periodic interval was calculated using the formula *K* = *N*/*n*, where *K* is a periodic interval, *N* is the total population, and *n* is the estimated sample size, accordingly, *K* = 1287/325; approximately, every fourth pregnant women attended ANC service during the data collection period was included in the study.

### 2.5. Data Collection Tools and Procedures

Data were collected by five trained midwives through a face-to-face interview. A structured questionnaire was developed through reviewing available relevant literature adapted from previous similar studies. The instrument includes items related to sociodemographic, antenatal depression, social support, obstetric and clinical parameters, intimate partner violence, and substance-related characteristics. Patient health questionnaire (PHQ-9) was used to screen antenatal depression symptom severity; accordingly, PHQ-9-score of 0-4 indicates minimal depression, 5-9 mild depression, 10-14 moderate depression, 15-19 moderately severe depression, and 20-27 as severe depression. A score of 10 and above is considered as a cutoff score for depression [23]. PHQ-9 has acceptable reliability and validity for the screening of antenatal depressive symptoms and for measuring the severity of depressive symptoms among Ethiopian pregnant women [24]. Maternity Social Support Scale (MSSS) was used to measure the respondent's social support status. The tool is a five-point Likert scale with a total maximum score of 30. Each item was rated as 5 for always, 4 for most of the time, 3 for some of the time, 2 for rarely, and 1 for never. Accordingly, social support was classified into three categories: high social support (scores 24–30), medium social support [18–23], and low social support (below 18) categories [21]. Intimate partner violence was measured by intimate partner violence scale; intimate partner violence was considered if a pregnant woman reported one of the following during the current pregnancy: husband/partner have done things to scare or intimidate on purpose or threatened to hurt someone they care about or hit, slapped, or thrown something that could hurt her or forced or pressured to have sexual intercourse when she did not want to [25, 26]. Obstetric and clinical parameters in this study include pregnancy planning, gravidity, parity, trimester, history of abortion, history of stillbirth, perceived complication during a previous pregnancy, having currently known medical illness, and currently taking medication. A family history of depression was assessed with the question: Is there any family member who has a diagnosis of depression? Substance use in this study includes frequent use of alcohol, khat (a local stimulant drug), tobacco, and cannabis. Moreover, substance use was measured by the WHO Alcohol, Smoking, and Substance Involvement Screening Test (ASSIST) screening tool. Accordingly, lifetime use was considered when a pregnant woman used any of the above-listed substances ever in her life, and the current use was considered when the pregnant woman used any of the above-listed substances within the last three months [27]. The English version of the questionnaire was translated into local language Amharic and Afaan Oromo and back translated into English to ensure its consistency by independent language experts of the field. Finally, the Amharic and Afaan Oromo versions of the questionnaire were used to collect the required data.

### 2.6. Quality Control Measures

Regular supervision was made by the data collection supervisors to ensure all necessary data were properly collected. Each day of data collection, the filled questionnaires were checked manually first for completeness and consistency then inserted into the computer twice. A pilot test was conducted to identify the impending problems on data collection tools on 5% of respondents at the Bocho-bore Health Center; the necessary correction of the questionnaire was done accordingly to the pilot test result; the data collected during pilot test were not included in the analysis of the main study.

### 2.7. Data Process, Analysis, and Presentation

The collected data were cleaned, coded, and entered into the Epi-data version 3.1 data entry software and exported to the Statistical Package for the Social Sciences (SPSS) version 20 statistical software for analysis. Descriptive statistics were done to summarize the dependent and independent variables. A chi-square test of association was performed to identify factors associated with the outcome variable. Finally, variables with a *P* value of less than 0.05 were considered to have a statistically significant association with the outcome variable. The final result was organized through text narration and table form.

### 2.8. Ethical Consideration

The study was reviewed and approved by the Institutional Review Board (IRB) of Jimma University. Furthermore, permission was obtained from the Kochi Health Center for data collection. Participants were fully informed about the aims of the study, and written informed consent was sought before the interview. Privacy and confidentiality were ensured throughout the process of the study.

This study was conducted in accordance with the Declaration of Helsinki.

## 3. Results

### 3.1. Sociodemographic Characteristics

A total of 314 of pregnant women gave their consent to participate in this study, resulting in a response rate of 96.7%. The majority of the respondents 121 (38.5%) were between the ages of 25 and 29 years old and were housewives 176 (56.1%). Most of the respondents 141 (44.9%) were Muslim, attended primary school 116 (36.9%), and earn less than one thousand birr (around 29 USD) monthly 171 (54.5%) (Table 1).

### 3.2. Clinical Characteristics of the Respondents

Nearly all 289 (92%) pregnancies were planned, and three-quarters of pregnant women were multigravida 230 (73.2%). Almost half of the women were on second trimester 158 (50.4%), and 11 (3.5%) had a history of abortion. Moreover, 41 (13%) reported perceived complications during a previous pregnancy, and 34 (10.8%) of the respondents were current substance abusers. Almost one-fifth of the respondents were reported having currently known medical illness, and among them, more than two-thirds 41 (70.7%) were currently taking medication (Table 2).

### 3.3. Social Support Status of the Respondent

Approximately one-third 94 (29.9%) of the total respondents reported as they have good friends who always support them, and nearly half of the participants described as their husband or partner who always helps them a lot. Additionally, two-thirds of the respondents were reported as having a conflict with their husband or partner sometimes 109 (34.6), and about 141 (44.9%) replied as feeling always controlled by their husband or partner (Table 3).

### 3.4. Intimate Partner Violence Status of the Respondent

A total of 53 (16.9%) of the study participants reported that their husband or partner has done things to scare or intimidate them, and nearly one-fifth 61 (19.4%) of the respondents described that they were hit, slapped, or thrown something that could hurt them by their husband or partner. Moreover, 16 (5.1%) reported that they were forced or pressured by their husband or partner to have sexual intercourse when they did not want to (Table 4).

### 3.5. Prevalence of Antenatal Depression

A total of 52 (16.6%) of the respondent had depression; among them, 35 (11.1%), 11 (3.5%), and 6 (1.9%) had moderate, moderately severe, and severe depression, respectively (Table 5).

### 3.6. Factors Associated with Antenatal Depression

A chi-square test of independence has revealed statistically significant association between antenatal depression and marital status, *X*^2^ (3, *N* = 314) =72.1353, *P* = <0.00001, and family history of depression, *X*^2^ (1, *N* = 314) =29.2109, *P* < 0.00001. Likewise, the analysis result has shown the association between antenatal depression and pregnancy planning, *X*^2^ (1, *N* = 314) =69.4507, *P* < 0.00001. Additionally, statistically significant association was found between antenatal depression and history of abortion, *X*^2^ (1, *N* = 314) = 26.0251, *P* < 0.00001; social support, *X*^2^ (2, *N* = 314) = 86.1944, *P* < 0.00001; and intimate partner violence, *X*^2^ (2, *N* = 314) = 41.5347, *P* < 0.00001 (Tables 6 and 7).

## 4. Discussion

This study was conducted to assess the prevalence and factors associated with antenatal depression among pregnant women attending ANC service in Kochi Health Center, Jimma, Ethiopia.

The study has revealed that 52 (16.6%) of the pregnant woman had antenatal depression during the current pregnancy. This result is nearly similar to 16% in the longitudinal study done in the US [28] but much lower than 31.9% in the study done in Saudi Arabia [29], 42.7% in Pakistan [30], and 15 to 65% in a systematic review done globally [31]. On the contrary, the finding of this study is higher than 9.18% and 12.3% in the study done in Mumbai and Bengaluru, India, respectively [32, 33], and 14.2% in Rio de Janeiro, Brazil [34]; this may be because a better health service provision of the countries compared to Ethiopia. The finding of this study is almost similar to 15.9% in Southern KwaZulu-Natal, South Africa [35], 17.9% in the Dubti Hospital Afar region [21], and 15.2% in Aneded woreda, Amhara Region, Ethiopia [36]. But the result is lower than 24.3% in a systematic review and meta-analysis in South Asia [37], 24.5% in Abeokuta North Local Government Area in Nigeria [38], 21.5% in the findings from, 21.28% in a systematic review and meta-analysis in Ethiopia [22], and 23.5% in another systematic review and meta-analysis in Ethiopia [8], Hawassa [39], and 24.9% in Addis Ababa [40]. The finding is much lower when it compared to 47% in rural South Africa [41] and 37.6% in Rwanda [42]; this variation may be explained by the difference in the socio-cultural-economic status of the countries, the difference in the study tool used, cutoff point scores, and the sample size used.

In this study, marital status was found to have a significant association with antenatal depression; this is similar with the studies done in Poland [43], Abeokuta North Local Government Area, Nigeria [38], Mombasa city, Kenya [44]; a systematic review and meta-analysis in Ethiopia [8]; another systematic review in Ethiopia [8]; and the national health survey Ethiopia [45], Anended woreda in Amhara Region Ethiopia [36], and Ankesha district, Awi zone, North West Ethiopia [46]. This could be because the psychological, physical, and economic support by the partner could play a significant role in reducing the risk of depression during pregnancy [47].

Similarly, family history of depression was associated with antenatal depression; this finding is similar with the studies done in Dare Salam Tanzania [48]; an umbrella review on the global burden of antenatal depression [31], the study was done in Arba Minch [49] and Mizan Aman [50]. This could be explained by the role of genetics and psychosocial support system of the family dynamics that could contribute to the development of depression during pregnancy [51].

In the current study, pregnancy planning was found to have an association with antenatal depression similarly; consistent findings were reported from the study done in Hyderabad, Pakistan [52], and Nigeria [38] and a systematic review in Ethiopia [8], Aneded woreda, North West Ethiopia [36], Addis Ababa [53], Hawassa [39], and Awi zone, North West Ethiopia [46]. This showed that psychological preparedness and readiness to conceive might be a contributing factor to depression during gestation.

In this study, social support was associated with antenatal depression; similar findings were reported from the study done in the USA [54], KwaZulu-Natal, South Africa [35], and Kenya [44]; a systematic review and meta-analysis conducted in Ethiopia [22]; and another systematic review in Ethiopia [8] and Hawassa, Ethiopia [39]. This might be explained by social support that has been shown to promote mental health and acts as a buffer against depression [55], so lack of social support could be implicated for the high risk of depression during pregnancy.

A significant association was found between intimate partner violence and antenatal depression; similar reports were obtained from the studies done in Hyderabad, Pakistan [52], and KwaZulu-Natal, South Africa [35]; a case-control study in Kenya [44]; and a systematic review and meta-analysis conducted in Ethiopia [22] and Aneded woreda, North West Ethiopia [36]. This could reveal that psychological, physical, and sexual abuse by the intimate partner particularly during pregnancy could lead to depressive symptomatology.

This study has found the association between history of abortion and antenatal depression; similar findings were reported from the study done in the US [56], Netherlands [57], China [58], Japan [59], and Hawassa [39]. Having a history of spontaneous abortion could have strong psychological sequelae. This will ultimately induce fear, uncertainty, worry, and anxiety and further increase the risk of depression during subsequent pregnancies [47].

## 5. Conclusions

The prevalence of antenatal depression among pregnant women attending ANC service in Kochi Health Center was high and significantly associated with marital status, family history of depression, pregnancy planning, history of abortion, social support, and intimate partner violence. Integration of mental health service with ANC follow-up service and screening pregnant women for possible depression has paramount importance for a healthy pregnancy and prevention of the possible adverse health outcomes on the mother and the fetus.

## Figures and Tables

**Table 1:** Sociodemographic characteristics of respondents in Kochi Health Center, Jimma town, southwest Ethiopia, 2019.

Variables	Categories	Frequency	Percentage
Residence	Urban	289	92.1
	Rural	25	7.9

Age of the respondents	15-19	23	7.4
	20-24	72	22.9
	25-29	121	38.5
	30-34	80	25.4
	≥35	18	5.8

Occupation	Housewife	176	56.1
	Government employee	32	10.2
	Merchant	56	17.8
	Daily laborer	28	8.9
	Others^∗^	22	7.0

Marital status	Single	19	6.1
	Married	280	89.2
	Divorced	11	3.5
	Widowed	4	1.2

Ethnicity	Oromo	149	47.5
	Kefa	73	23.2
	Amhara	61	19.4
	Yem	25	8.0
	Others^∗^	6	1.9

Religion	Muslim	141	44.9
	Orthodox	96	30.6
	Protestant	60	19.1
	Others^∗^	17	5.4

Educational status	Illiterate	57	18.1
	Read and write	65	20.7
	Primary school	116	36.9
	Secondary school	51	16.2
	College and above	25	8.0

Family monthly income	<1000 birr	171	54.5
	1000-2500 birr	90	28.6
	>2500 birr	53	16.9

^∗^Other occupation includes waiters and homemade. ^∗^Other ethnicities include Gurage, Tigre, and Wolayeta. ^∗^Other religion includes catholic and Wakefeta.

**Table 2:** Mental, obstetric, and medical characteristics of respondents in Kochi Health Center, Jimma town, southwest Ethiopia, 2019.

Variables	Categories	Frequency	Percentage
Past history of diagnosed mental illness	Yes	21	6.6
	No	293	93.4

Family history of depression	Yes	35	11.1
	No	279	88.6

Pregnancy planning	Planned	289	92.0
	Unplanned	25	8.0

Gravidity	Primigravida	84	26.8
	Multigravida	230	73.2

Parity	None	84	26.8
	One	98	31.2
	Two or more	132	42.0

Trimester	First	62	19.7
	Second	158	50.4
	Third	94	29.9

History of abortion	Yes	11	3.5
	No	303	96.5

History of stillbirth	Yes	23	7.3
	No	291	92.7

Perceived complication during a previous pregnancy	Yes	41	13.0
	No	273	87.0

Substance use	No	100	31.9
	Lifetime user	180	57.3
	Current user	34	10.8

Current substance use type	Alcohol	17	50.0
	Khat	12	35.2
	Tobacco	4	11.8
	Cannabis	1	3.0

Having currently known medical illness	Yes	58	18.5
	No	256	81.5

Currently taking medication	Yes	41	70.7
	No	17	29.3

**Table 3:** Social support of the respondents in Kochi Health Center, Jimma town, southwest Ethiopia, 2019.

Category	Frequency (%)				
How do you feel about the support you have right now?	Always	Most of the time	Some of the time	Rarely	Never
I have good friends who support me	94 (29.9)	82 (26.1)	58 (18.5)	42 (13.4)	38 (12.1)
My family is always there for me	156 (49.7)	96 (30.5)	32 (10.2)	21 (6.7)	9 (2.9)
My husband or partner helps me a lot	143 (45.5)	98 (31.2)	36 (11.5)	25 (8.0)	12 (3.8)
There is a conflict with my husband or partner	43 (13.7)	51 (16.2)	109 (34.6)	58 (18.7)	53 (16.8)
I feel controlled by my husband or partner	141 (44.9)	121 (38.5)	33 (10.6)	11 (3.5)	8 (2.5)
I feel loved by my husband or partner	89 (28.3)	113 (36.0)	67 (21.3)	23 (7.4)	22 (7)

**Table 4:** Intimate partner violence of the respondents in Kochi Health Center, Jimma town, southwest Ethiopia, 2019.

Variables	Categories	Frequency		Percentage
During your present pregnancy, has your husband or partner:	Done things to scare or intimidate you on purpose	Yes	53	16.9
		No	261	83.1
	Threatened to hurt you or someone you care about	Yes	14	4.5
		No	300	95.5
	Hit you, slapped you, or thrown something at you that could hurt you	Yes	61	19.4
		No	253	80.6
	Forced you or pressured you to have sexual intercourse when you did not want to	Yes	16	5.1
		No	298	94.9

**Table 5:** Prevalence of antenatal depression among pregnant women attending Kochi Health Center, Jimma town, southwest Ethiopia, 2019.

Depression severity	Frequency	Percentage
Minimal	149	47.5
Mild	113	36.0
Moderate	35	11.1
Moderately severe	11	3.5
Severe	6	1.9
Total	314	100

**Table 6:** Factors associated with antenatal depression among pregnant women in Kochi Health Center, Jimma town, southwest Ethiopia, 2019.

Variables	Categories	Frequency (%)	Antenatal depression among pregnant woman		Chi-square (*P* value)
			Yes (%)	No (%)	
Residence	Urban	289 (92.1)	50 (17.3)	239 (82.7)	1.4405 (0.230052)
	Rural	25 (7.9)	2 (8)	23 (92)	

Age of the respondents	15-19	23 (7.4)	4 (17.4)	19 (82.6)	6.9594 (0.138052)
	20-24	72 (22.9)	16 (22.2)	56 (77.8)	
	25-29	121 (38.5)	23 (19)	98 (81)	
	30-34	80 (25.4)	6 (7.5)	74 (92.5)	
	≥35	18 (5.8)	3 (16.7)	15 (83.3)	

Occupation	Housewife	176 (56.1)	25 (14.2)	151 (85.6)	4.2657 (0.371246)
	Government employee	32 (10.2)	5 (15.6)	27 (84.4)	
	Merchant	56 (17.8)	9 (16.1)	47 (83.9)	
	Daily laborer	28 (8.9)	8 (28.6)	20 (71.4)	
	Others^∗^	22 (7.9)	5 (22.7)	17 (77.3)	

Marital status	Single	19 (6.1)	15 (79.0)	4 (21.1)	72.1353 (< 0.00001)^∗^
	Married	280 (89.2)	30 (10.7)	250 (89.3)	
	Divorced	11 (3.5)	6 (54.5)	5 (45.4)	
	Widowed	4 (1.2)	1 (25)	3 (75)	

Ethnicity	Oromo	149 (47.5)	26 (17.5)	123 (82.5)	1.6634 (0.797358)
	Kefa	73 (23.2)	10 (13.7)	63 (86.3)	
	Amhara	61 (19.4)	9 (14.8)	52 (85.2)	
	Yem	25 (8.0)	6 (24)	19 (76)	
	Others^∗^	6 (1.9)	1 (16.7)	5 (83.3)	

Religion	Muslim	141 (44.9)	24 (17.0)	117 (83.0)	0.4443 (0.930957)
	Orthodox	96 (30.6)	14 (14.6)	82 (85.4)	
	Protestant	60 (19.1)	11 (18.3)	49 (81.7)	
	Others^∗^	17 (5.4)	3 (17.6)	14 (82.4)	

Educational status	Illiterate	57 (18.1)	10 (17.5)	47 (82.5)	8.2355 (0.083324)
	Read and write	65 (20.7)	18 (27.7)	47 (72.3)	
	Primary school	116 (36.9)	14 (12.1)	102 (87.9)	
	Secondary school	51 (16.2)	7 (13.7)	44 (86.3)	
	College and above	25 (8.0)	3 (12.0)	22 (88.0)	

Family monthly income	<1000 birr	171 (54.5)	27 (15.8)	144 (84.2)	0.1769 (0.915365)
	1000-2500 birr	90 (28.6)	16 (17.8)	74 (82.2)	
	>2500 birr	53 (16.9)	9 (17)	44 (83)	

^∗^Other occupation includes waiters and homemade. ^∗^Other ethnicities include Gurage, Tigre, and Wolayeta. ^∗^Other religion includes catholic and Wakefeta.

**Table 7:** Factors associated with depression among pregnant women in Kochi Health Center, Jimma town, southwest Ethiopia, 2019.

Variables	Categories	Frequency (%)	Antenatal depression among pregnant woman		Chi-square (*P* value)
			Yes (%)	No (%)	
Past history of diagnosed mental illness	Yes	21 (6.6)	4 (19.1)	17 (80.9)	0.1007 (0.750937)
	No	293 (93.4)	48 (16.4)	245 (83.6)	

Family history of depression	Yes	35 (11.1)	17 (48.6)	18 (51.4)	29.2109 (< 0.00001)^∗^
	No	279 (88.6)	35 (12.5)	244 (87.5)	

Pregnancy planning	Planned	289 (92.0)	33 (11.4)	256 (88.6)	69.4507 (< 0.00001)^∗^
	Unplanned	25 (8.0)	19 (76)	6 (24)	

Gravidity	Primigravida	84 (26.8)	14 (16.7)	70 (83.3)	0.0009 (0.975603)
	Multigravida	230 (73.2)	38 (16.5)	192 (83.5)	

Parity	None	84 (26.8)	14 (16.7)	70 (83.3)	1.3114 (0.519084)
	One	98 (31.2)	13 (20.4)	85 (79.6)	
	Two or more	132 (42.0)	25 (6.9)	107 (83.1)	

Trimester	First	82 (19.7)	14 (37.1)	68 (62.9)	2.523 (0.283236)
	Second	138 (50.4)	27 (11.4)	111 (88.6)	
	Third	94 (29.9)	11 (11.7)	83 (88.3)	

History of abortion	Yes	11 (3.5)	8 (72.8)	3 (26.2)	26.0251 (<0.00001)^∗^
	No	303 (96.5)	44 (14.5)	259 (85.5)	

History of stillbirth	Yes	23 (7.3)	6 (26.1)	17 (73.9)	1.63 (0.201706)
	No	291 (92.7)	46 (15.8)	245 (84.2)	

Perceived complication during a previous pregnancy	Yes	41 (13.0)	8 (46.3)	33 (53.7)	0.2973 (0.585558)
	No	273 (87.0)	44 (12.1)	229 (87.9)	

Substance use	No	100 (31.9)	13 (13.0)	87 (87.0)	5.8021 (0.054965)
	Lifetime user	180 (57.3)	37 (20.6)	143 (79.4)	
	Current user	34 (10.8)	2 (5.9)	32 (94.1)	

Having currently known medical illness	Yes	58 (18.5)	13 (22.4)	45 (77.6)	1.7639 (0.18414)
	No	256 (81.5)	39 (15.2)	217 (84.8)	

Currently taking medication	Yes	41 (70.7)	13 (31.7)	28 (68.3)	0.0702 (0.791065)
	No	17 (29.3)	6 (35.3)	11 (64.7)	

Social support	High	21 (6.7)	9 (42.9)	12 (57.1)	86.1944 (< 0.00001)^∗^
	Medium	258 (82.2)	20 (6.8)	238 (93.2)	
	Low	35 (11.1)	23 (65.7)	12 (34.3)	

Intimate partner violence	Present	144 (88.6)	45 (31.2)	99 (68.8)	41.5347 (<0.00001)^∗^
	Absent	170 (92.0)	7 (4.1)	163 (95.9)	

^∗^Variables which showed significant association at *P* value <0.05 in chi-square analysis.

## Data Availability

The datasets used and/or analyzed during the current study are available from the corresponding author on reasonable request.
